# Diagnosis of Non-Hepatocellular Carcinoma Malignancies in Patients With Risks for Hepatocellular Carcinoma: CEUS LI-RADS Versus CT/MRI LI-RADS

**DOI:** 10.3389/fonc.2021.641195

**Published:** 2021-04-12

**Authors:** Yi-Xin Hu, Jing-Xian Shen, Jing Han, Si-Yue Mao, Ru-Shuang Mao, Qing Li, Fei Li, Zhi-Xing Guo, Jian-Hua Zhou

**Affiliations:** ^1^ Department of Ultrasound, Sun Yat-sen University Cancer Center, State Key Laboratory of Oncology in South China, and Collaborative Innovation Center for Cancer Medicine, Guangzhou, China; ^2^ Image and Minimally Invasive Intervention Center, Sun Yat-sen University Cancer Center, State Key Laboratory of Oncology in South China, and Collaborative Innovation Center for Cancer Medicine, Guangzhou, China

**Keywords:** contrast-enhanced ultrasound, computed tomography, magnetic resonance imaging, liver imaging reporting and data system, non-hepatocelluar carcinoma malignancies

## Abstract

**Objective:**

Data regarding direct comparison of contrast-enhanced ultrasound (CEUS) Liver Imaging Reporting and Data System (LI-RADS) and Computed Tomography/Magnetic Resonance Imaging (CT/MR) LI-RADS in diagnosis of non-hepatocelluar carcinoma (non-HCC) malignancies remain limited. Our study aimed to compare the diagnostic performance of the CEUS LI-RADS version 2017 and CT/MRI LI-RADS v2018 for diagnosing non-HCC malignancies in patients with risks for HCC.

**Materials and Methods:**

In this retrospective study, 94 liver nodules pathologically-confirmed as non-HCC malignancies in 92 patients at risks for HCC from January 2009 to December 2018 were enrolled. The imaging features and the LI-RADS categories on corresponding CEUS and CT/MRI within 1 month were retrospectively analyzed according to the ACR CEUS LI-RADS v2017 and ACR CT/MRI LI-RADS v2018 by two radiologists in consensus for each algorithm. The sensitivity of LR-M category, inter-reader agreement and inter-modality agreement was compared between these two standardized algorithms.

**Results:**

Ninety-four nodules in 92 patients (mean age, 54 years ± 10 [standard deviation] with 65 men [54 years ± 11] and 27 women [54 years ± 8]), including 56 intrahepatic cholangiocarcinomas, 34 combined hepatocellular cholangiocarcinomas, two adenosquamous carcinomas of the liver, one primary hepatic neuroendocrine carcinoma and one hepatic undifferentiated sarcoma were included. On CEUS, numbers of lesions classified as LR-3, LR-4, LR-5 and LR-M were 0, 1, 10 and 83, and on CT/MRI, the corresponding numbers were 3, 0, 14 and 77. There was no significant difference in the sensitivity of LR-M between these two standardized algorithms (88.3% of CEUS vs 81.9% of CT/MRI, p = 0.210). Seventy-seven lesions (81.9%) were classified as the same LI-RADS categories by both standardized algorithms (five for LR-5 and 72 for LR-M, kappa value = 0.307). In the subgroup analysis for ICC and CHC, no significant differences were found in the sensitivity of LR-M category between these two standardized algorithms (for ICC, 94.6% of CEUS vs 89.3% of CT/MRI, p = 0.375; for CHC, 76.5% of CEUS vs 70.6% of CT/MRI, p = 0. 649).

**Conclusion:**

CEUS LI-RADS v2017 and CT/MRI LI-RADS v2018 showed similar value for diagnosing non-HCC primary hepatic malignancies in patients with risks.

## Introduction

Primary hepatic malignancies consist of HCC (75–85%) (1) and non-HCC malignancy, which includes intrahepatic cholangiocarcinoma (ICC, 10–15%) (1), combined hepatocellular cholangiocarcinoma (CHC, <1%) (2) and other rare malignancies. Biological behaviors of HCC and non-HCC malignancy are sharply different, resulting as different optimal treatments and clinical prognoses. For example, liver transplantation, the first-line therapy for transplantable HCC in several countries (3), is not recommended for non-HCC malignancies due to relatively poor long-term outcomes in most institutions (2, 4). Moreover, non-HCC malignancy, mostly ICC, has a high potential for metastases, of which five-year survival is even worse than for HCC (5). Thus, it is very important to distinguish non-HCC from HCC to improve overall survival.

HCC may be noninvasively diagnosed by imaging findings alone, often without biopsy (6, 7). To standardized imaging and reporting, the American College of Radiology (ACR) developed LI-RADS at CT or MRI in patients at risks for HCC in 2011, which has been refined and expanded over multiple updates to version 2018 till now to consist with clinical practice, such as introducing the concept of LR-OM for non-HCC malignancies in 2013, which was renamed as LR-M in 2014 (8, 9). Besides, CEUS highlights itself with a real-time observation (10), for which ACR established the CEUS LI-RADS in 2016 (11) and further revised LR-M observations in 2017 (8, 9, 12). Several studies convinced the value of LR-M observation for differentiating non-HCC malignancies from HCC. An et al. (13) found that CT and MRI showed comparable capabilities for distinguishing non-HCC malignancies from HCC based on CT/MRI LI-RADS, with pooled accuracies of 79.9 and 82.4% for categorizing LR-M, respectively (p = 0.139). Kim et al. (14) demonstrated that non-HCC malignancy could be distinguished from HCC at a sensitivity of 89% and a specificity of 48% in patients with liver cirrhosis by using the LR-M criteria of CT/MRI LI-RADS v2018 at gadoxetate-enhanced MRI. Zheng et al. (15) validated the CEUS LI-RADS by showing a sensitivity of 89% and a specificity of 88% for the LR-M category to distinguish non-HCC malignancy from HCC.

Due to good diagnostic performance, CT/MRI LI-RADS was integrated into HCC guidance from American Association for the Study of Liver Diseases (AASLD) in 2018 (6). However, AASLD does not accept CEUS as a diagnostic technique but a second-line technique after CT or MRI for HCC due to the possibility that ICC may be misdiagnosed as HCC by displaying vascular patterns similar to HCC on CEUS (16). In order to maintain the specificity of LR-5 for HCC, the category of LR-M is intended to encompass non-HCC malignancies as much as possible (17). Previous studies have showed no significant difference in the specificity of LR-5 for HCC between CEUS LI-RADS and CT/MR LI-RADS (93.8% of CEUS vs 83.3% of CT/MRI, p = 0.109) (18), however, the sensitivity of LR-M for non-HCC malignancies between these two algorithms has not been fully evaluated due to the limited cases of non-HCC malignancies (18, 19). Thus, our study aimed to compare the sensitivity of LR-M for non-HCC primary hepatic malignancies in patients with risks and evaluate the inter-modality agreement between these two standardized algorithms with large sample of non-HCC malignancies.

## Materials And Methods

**Figure 1:**
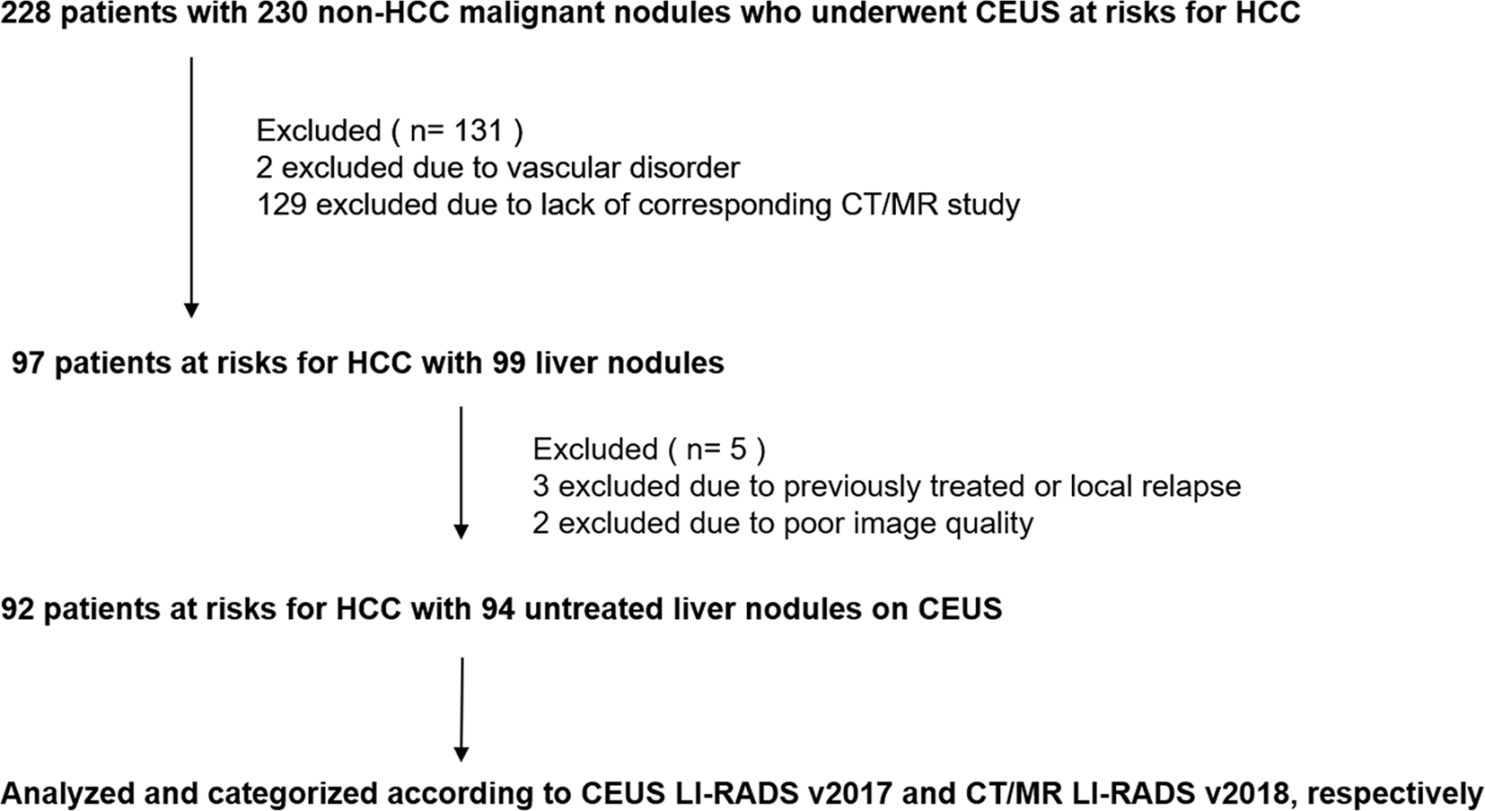
Flowchart of study sample.CEUS, contrast enhanced ultrasound; HCC, hepatocellular carcinoma; LI-RADS, Liver Imaging Reporting and Data System.

This retrospective study was approved by our institutional review board. The requirement to obtain written informed patient consent was waived. A flow diagram of our study population is shown in **Figure 1**. Twenty-nine and thirty-three patients enrolled in the present study were previously published by W.Z. (15) and F.L. (20), respectively. One aimed to evaluate the diagnostic performance of CEUS LI-RADS v2017 for all kinds of liver neoplasms (15), the other aimed to evaluate diagnostic performance of CEUS LI-RADS v2017 in distinguishing HCC from ICC (20), rather than comparing performance of the two algorithms in only non-HCC lesions in the current study.

### Study Population

Between January 2009 and December 2018, we retrospectively identified 228 patients with risks of HCC who had liver nodules confirmed as non-HCC malignancies and underwent CEUS at our institution before surgery or biopsy. Criteria of inclusion and exclusion were as followed. Finally, 94 lesions in 92 patients (mean age, 54 years; standard deviation, 10; range, 29–74 years) were enrolled.

The inclusion criteria were (a) age 18 years or older; (b) chronic hepatitis B viral infection, or pathologically-proven cirrhosis; (c) availability of corresponding CEUS and CT/MRI study within 1 month; and (d) availability of precise pathological results as non-HCC malignancies.

The exclusion criteria were (a) cirrhosis due to congenital hepatic fibrosis or a vascular disorder, such as hereditary hemorrhagic telangiectasia; (b) previously-treated or local-relapsed lesions; and (c) poor image quality or no complete series of imaging at each phase for each nodule.

### Image Acquisition

#### Conventional US and CEUS

Conventional US and CEUS data were acquired with Acuson or Acuson Sequoia 512 system (Siemens Medical Solutions, Mountain View, CA) equipped with a 4C1 convex array probe transducer. The size and echogenicity of the tumor on gray-scale US, were recorded. CEUS was performed with low-mechanicalindex (mainly 0.15–0.19) and dynamic range of 80 dB after the administration of 2.0 ml bolus of SonoVue (Bracco Imaging, Milan, Italy) in the antecubital vein followed by a flush of 5-ml saline. Imaging was continuously recorded on cine clips for 60 s immediately after contrast agent administration followed by intermittent scanning with 10–20 s at each interval for at least 5 min, without any change in the machine settings. The imaging data were divided into arterial (10–30 s), portal (30–120 s), and late phases (>120 s).

#### CT/MRI

Dynamic CT was performed at 64-section spiral CT system (Aquilion TSX-101A, Toshiba Medical Systems; LightSpeed 16, GE Health Care) with both 1- and 5-mm section thicknesses in 38 of 92 patients, who received administration of non-ionic iodine contrast medium (Ultravist; Iopromide; Schering) at a rate of 3 ml per second for patients (dose of 1.5 ml/kg of body weight). Dynamic CT was initiated at 30–40 s (hepatic arterial phase) and 55–60 s (portal venous phase) after contrast injection.

MRI image was acquired in the remaining 54 patients with a 3.0T system (Discovery MR750, GE Healthcare; Magnetom Trio Tim, Siemens Medical Solutions),. All patients underwent T1-weighted in-phase (450/15 repetition time/echo time [TR/TE], 224 × 256 matrix, 45× flip angle) and out-of phase gradient echo, slice thickness of 8 mm and transverse T2-weighted breath-hold, slice thickness of 5 mm (2,000/120 [TR/TE], 320 × 320 matrix, 120× flip angle) and dynamic enhanced scanning. DWI was performed with B value of 800 s/mm^2^ and slice thickness of 5 mm. Thirty-seven patients received administration of intravenous injection of gadopentetate dimeglumine (Magnevist; Schering, Germany) at the rate of 2 ml per second for patients (dose of 0.2 mmol/kg of body weight). The other 17 patients received administration of intravenous injection of gadoxetate disodium (Primovist, Bayer Pharma) at the rate of 2 ml per second for patients (dose of 0.025 mmol/kg of body weight), followed by a flush of 20 ml saline. Images are acquired at 30–40 s (arterial phase), 55–60 s (portal phase) and 3 min (delayed venous phase for gadopentetate; transitional phase [TP] for gadoxetate) as well as 15 and 20 min (hepatobiliary phase [HBP] for gadoxetate) after contrast injection.

### Image Analysis

#### CEUS

Two investigators (F.L. and J.H. with 4 and 7 years of experience respectively in hepatic imaging and CEUS) performed all categorization given according to the CEUS-LIRADS v2017. Images were reevaluated together until a consensus was reached. Other imaging results, including results of CT/MRI image analysis, and pathologic results were blinded to the reviewers. An explanation of each major features is listed in **Table 1**.

**Table 1:** Explanation of Each Imaging Feature for LR-M and Category.

Feature/Category	Definition
**CEUS LI-RADS**	
Rim APHE	Arterial phase enhancement is most pronounced in observation periphery
Early washout	Whole or partial hypoenhancement relative to liver occurs within 60 s after injection of the contrast agent.
Marked washout	Nodule becomes markedly hypoenhanced (appears as black hole) within 2 min (otherwise defined as mild)
**CT/MR LI-RADS**	
Rim APHE	Arterial phase enhancement that is most pronounced in observation periphery
Peripheral washout	An apparent washout that is most pronounced in observation periphery
Delayed central enhancement	Central area of progressive postarterial phase enhancement
Targetoid restriction	Concentric pattern on DWI characterized by restricted diffusion in observation periphery with less restricted diffusion in observation center
Targetoid TP or HBP appearance	Concentric pattern in TP or HBP characterized by moderate-to-marked hypointensity in observation periphery with milder hypointensity in center
**Categories of LI-RADS**	
LR-1	100% certainty that the finding is benign
LR-2	Probably benign
LR-3	Intermediate malignancy probability
LR-4	Probably HCC
LR-5	Definitely HCC
LR-M	Probably or definitely malignant but not HCC specific
LR-TIV	Definite tumor in vein

CEUS, contrast enhanced ultrasound; LI-RADS, Liver Imaging Reporting and Data System; APHE, arterial phase hyperenhancement; TP, transitional phase; HBP, hepatobiliary phase.

#### CT/MR

Two radiologists (J.X.S and S.Y.M. with 20 and 5 years of experience in CT/MRI scan of liver) analyzed CT/MRI images following CT/MRI-LI-RADS v2018. Images were reevaluated together until a consensus was reached. Other imaging results, such as results of CEUS image analysis, and pathologic results were blinded to the reviewer. An explanation of each major features assessed is listed in **Table 1**.

### Statistical Analysis

Statistical analysis was performed with software (Microsoft Excel 2019, Microsoft; SPSS version 20.0 for windows; SPSS, Inc, Chicago, IL, USA). Descriptive analysis was reported as absolute numbers and rates in percentages. Continuous variables were expressed as medians or means and ranges and were compared by using Student t test. The sensitivities of LR-M category of the two algorithms were compared by using the McNemar test and the inter-reader agreement between the two radiologists of each algorithm as well as the inter-modality agreement of the lesion classifications was analyzed by using Cohen k statistics as follows: k ≤0.00 indicated poor agreement; k = 0.01–0.20, slight agreement; k = 0.21–0.40, fair agreement; k = 0.41–0.60, moderate agreement; k = 0.61–0.80, substantial agreement; and k = 0.81–0.99, almost perfect agreement. A two-sided P value of less than 0.05 was considered to indicate a statistically significant difference.

## Results

### Participants and Lesions

Ninety-four non-HCC primary hepatic malignancies confirmed by pathology in 92 patients (mean age, 54 years ± 10 [standard deviation] with 65 men [54 years ± 11] and 27 women [54 years ± 8]) with risks were enrolled in the final analysis, including 56 (60%) intrahepatic cholangiocarcinomas, 34 (36%) combined hepatocellular cholangiocarcinomas, two (2%) adenosquamous carcinomas of the liver, one (2%) primary hepatic neuroendocrine carcinoma and one (1%) hepatic undifferentiated sarcoma. All patients enrolled in the final analysis were with chronic HBV infection, but without cirrhosis caused by other reasons. The characteristics of patient and nodule are shown in **Table 2**.

**Table 2:** Patient and Nodule Characteristics.

Variable	Value
**Patients (n = 92)**	
Male/Female (n)	65 (71)/27 (29)
Mean age (y)	54 ± 10
**Nodule size (n = 94)**	
<30 mm	23 (24)
≥30 mm	71 (76)
Median, range (mm)	42, 11–118
Mean (mm)	49 ± 25
**Number of nodules:1/2/>2**	90/2/0
**Histologic features**	
Intrahepatic cholangiocarcinoma	56 (60)
Combined hepatocellular cholangiocarcinoma	34 (36)
Adenosquamous carcinoma of the liver	2 (2)
Primary hepatic neuroendocrine carcinoma	1 (1)
Hepatic undifferentiated sarcoma	1 (1)
**Histologic confirmation method**	
Resection	85 (90)
Percutaneous biopsy	9 (10)
**Median AFP (range, ng/ml)**	4.91 (1.04–33,341)

Data are numbers (%) of patients and nodule, means ± SD, or medians (ranges).

### Imaging Features

On B-mode US, hypoecho was found in 72 lesions (76.6%); isoecho was found in nine lesions (9.6%); hyperecho was found in 13 lesions (13.8%).

As for size measurement of each nodule on US and CT/MRI, there was no significant difference in longest-axis measurement between CEUS and CT/MR (mean, 52 mm ± 27 [standard deviation] of CEUS vs 49 mm ± 25 of CT/MRI, p = 0.121). However, we found that there were 6 nodules divided into different categories (size < 20 mm vs size ≥20 mm), while divided nodule size at the cut-off size of 20 mm (kappa value = 0.466, p <0.001).

On CEUS, the frequencies of CEUS features on the LR-M nodules are summarized in **Table 3**, rim-like arterial phase hyperenhancement (rim APHE), early washout (<60 s), marked washout (<2 min) were respectively seen in 27 (28.7%), 82 (87.2%) and 15 (16.0%) of all 94 non-HCC malignancies. In consideration of precise histological results of the lesions, rim APHE, early washout (<60 s), marked washout (<2 min) were found in 8 (23.5%), 25 (73.5%), 3(8.8%) of 34 CHC, respectively, while the corresponding numbers were 17 (30.4%), 53 (94.6%), 10 (17.9%) of 56 ICC, respectively. After dividing all 94 lesions into two subgroups at the cut-off value of 30 mm for the nodule size, there was significant difference in the rate of feature occurring between the two subgroups (For rim APHE, 8.7% of lesions size <30 mm vs 35.2% of lesions size ≥30 mm, p = 0.015; for early-onset washout, 69.6% of lesions size <30 mm vs 93.0% of lesions size ≥30 mm, p =0.003). Similar results were found in 34 CHC (For rim APHE, 7.7% of lesions size <30 mm vs 33.3% of lesions size ≥30 mm, p = 0.087; for early-onset washout, 53.8% of lesions size <30 mm vs 85.7% of lesions size ≥30 mm, p = 0.041). However, there was no significant difference in the frequency of these three features found between the two subgroups in 56 ICC (p >0.05). More details were listed in **Table 3**.

**Table 3:** Specific Features of LR-M Nodules Based on Histologic Features and Nodules size on Contrast-enhanced US and CT/MRI.

	All lesion				CHC				ICC>			
**CEUS**	Total (n = 94)	<30 mm (n = 23)	≥30 mm (n = 71)	P value*	Total (n = 34)	<30 mm (n = 13)	≥30 mm (n = 21)	P value*	Total (n = 56)	<30 mm (n = 8)	≥30 mm (n = 48)	P value*
Rim arterial phase hyperenhancement	27 (29)	2 (9)	25 (35)	0.015	8 (24)	1 (8)	7 (33)	0.087	17 (30)	1 (13)	16 (33)	0.325
Early-onset washout (<60 s)	82 (87)	16 (69)	66 (93)	0.003	25 (74)	7 (54)	18 (86)	0.041	53 (95)	7 (88)	46 (96)	0.332
Marked washout (<2 min)	15 (16)	2 (9)	13 (18)	0.274	3 (9)	1 (8)	2 (10)	0.855	10 (18)	1 (13)	9 (19)	0.669
**CT/MR**	Total (n = 94)	<30 mm (n = 23)	≥30 mm (n = 71)	P value*	Total (n = 34)	<30 mm (n= 13)	≥30 mm (n = 21)	P value*	Total (n = 56)	<30 mm (n = 8)	≥30 mm (n = 48)	P value*
Rim arterial phase hyperenhancement	56 (60)	8 (35)	48 (68)	0.005	15 (44)	4 (31)	11 (52)	0.380	39 (70)	4 (50)	35 (73)	0.374
Delayed central enhancement	61 (65)	12 (52)	49 (69)	0.141	19 (56)	3 (23)	16 (76)	0.004	39 (70)	5 (63)	34 (71)	0.953
Peripheral washout	32 (34)	3 (13)	29 (41)	0.028	8 (24)	2 (15)	6 (29)	0.444	22 (39)	1 (13)	21 (44)	0.199
**MRI**	Total (n =54)	<30 mm (n = 13)	≥30 mm (n = 41)	P value*	Total (n = 22)	<30 mm (n = 8)	≥30 mm (n = 14)	P value*	Total (n = 31)	<30 mm (n = 5)	≥30 mm (n = 26)	P value*
DWI targetoid restriction	22 (41)	3 (23)	19 (46)	0.245	5 (23)	1 (13)	4 (29)	0.613	17 (55)	2 (40)	15 (58)	0.636
**Gadoxetate disodium-enhanced MRI**	Total (n= 17)	<30 mm (n = 4)	≥30 mm (n = 13)	P value*	Total (n = 6)	<30 mm (n = 3)	≥30 mm (n = 3)	P value*	Total (n = 11)	<30 mm (n = 1)	≥30mm (n= 10)	P value*
Targetoid transitional phase appearance	11 (65)	3 (75)	8 (62)	1.000	4 (67)	3 (100)	1 (33)	0.400	7 (64)	0 (0)	7 (70)	0.364
Targetoid hepatobiliary phase appearance	13 (77)	3 (75)	10 (77)	1.000	5 (83)	3 (100)	2 (67)	1.000	8 (73)	0 (0)	8 (80)	0.273

Data are numbers of observations, with percentages in parentheses. CT, computed tomography; MR, magnetic resonance imaging; CEUS, contrast enhanced ultrasound; ICC, intrahepatic cholangiocarcinomas; CHC, combined hepatocellular cholangiocarcinomas; *P values were determined with x^2^ test for comparison of the rate of feature occurring between the two subgroups divided according to the nodule size at the cut-off value of 30 mm.

For CT/MRI, rim APHE and delayed central enhancement, peripheral washout, were respectively seen in 56 (59.5%), 61 (64.9%), 32 (34.0%) of all 94 lesions. DWI targetoid restriction, targetoid TP appearance and targetoid HBP appearance were respectively found in 22 (40.7%) of 54 lesions on MRI and 11 (64.7%) and 13 (76.5%) of 17 lesions on Gadoxetate disodium-enhanced MRI.

### Diagnostic Performances

On CEUS, numbers of lesions classified as LR-3, LR-4, LR-5 and LR-M were 0, 1, 10 and 83, respectively, while on CT/MRI, the corresponding numbers were 3, 0, 14 and 77. There was no significant difference in the sensitivity of LR-M category between these two standardized algorithms (88.3% of CEUS vs 81.9% of CT/MRI, p = 0.210). Seventy-seven lesions (81.9%) were classified as the same LI-RADS categories on both guidelines (5 for LR-5 and 72 for LR-M, kappa value = 0.307, p = 0.001) (**Table 4**).

**Table 4:** Comparison of CEUS LI-RADS and CT/MRI LI-RADS category.

	CT/MRI				CEUS				P value*	kappa value^†^
	LR-3	LR-4	LR-5	LR-M	LR-3	LR-4	LR-5	LR-M		
**All lesions** **(n = 94)**	3 (3)	0 (0)	14 (15)	77 (82)	0 (0)	1 (1)	10 (11)	83 (88)	0.210	0.307
**ICC** **(n = 56)**	3 (5)	0 (0)	3 (5)	50 (90)	0 (0)	0 (0)	3 (5)	53 (95)	0.375	0.296
**CHC** **(n = 34)**	0 (0)	0 (0)	10 (29)	24 (71)	0 (0)	1 (3)	7 (21)	26 (76)	0.754	0.264

Data are numbers of observations, with percentages in parentheses. CT/MRI, Computed Tomography/Magnetic Resonance Imaging; CEUS Contrast Enhanced Ultrasound; ICC, intrahepatic cholangiocarcinomas; CHC, combined hepatocellular cholangiocarcinomas; *P values were determined with McNemar test for comparison of the rate of the lesions correctly classified as LR-M category between CEUS LI-RADS and CT/MRI LI-RADS.

^†^kappa values were determined with Cohen k statistics for comparison of CEUS LI-RADS and MRI-LI-RADS category.

Considering the histopathologic details of the liver lesions, one 24-mm CHC, which presented iso-enhancement at arterial phase with mild wash out onset at 2–3 min, was classified as LR-4, 7 CHC and 3 ICC were misdiagnosed as LR-5, and other 83 non-HCC were correctly classified as LR-M on CEUS; while, on CT/MR, three ICC was mistaken as LR-3, 10 CHC, 3 ICC and 1 hepatic neuroendocrine carcinoma were classified as LR-5, and other 77 non-HCC were correctly classified as LR-M.

**Figure 2:**
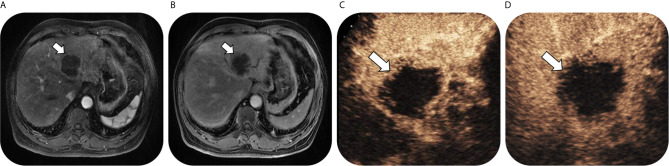
Images in a 63-year-old man with chronic hepatitis B virus infection and pathological confirmed intrahepatic cholangiocarcinoma lesion, which was correctly classified as LR-M both on CEUS and gadopentetate-enhanced MRI. T1-weighted image shows a 58-mm nodule in hepatic segment II/III/IV with rim arterial phase hyperenhancement (arrow) in **(A)** arterial phase followed by delayed central enhancement (arrow) in **(B)** portal phase. Contrast-enhanced US image shows a 68-mm nodule with rim arterial phase hyper-enhancement (arrow) in **(C)** arterial phase (timer, 00:22) followed by marked washout (arrow) visible in **(D)** portal phase (timer, 01:49).

**Figure 3:**
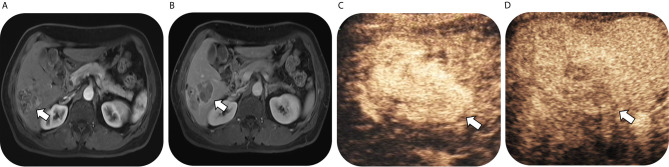
Images in a 54-year-old man with chronic hepatitis B virus infection and pathological confirmed combined hepatocellular cholangiocarcinoma, which was correctly classified as LR-M on CEUS but mistaken as LR-5 on gadopentetate-enhanced MRI. T1-weighted image shows a 53-mm nodule in hepatic segment VI with arterial phase hyperenhancementand (arrow) in **(A)** arterial phase followed by enhancing capsule (arrow) and non-rim washout in **(B)** delayed phase. Contrast-enhanced US image shows a 64-mm nodule with heterogeneous hyperenhancement (not rim or peripheral discontinued globular enhancement) (arrow) in **(C)** arterial phase (timer, 00:19) followed by early washout (arrow) in **(D)** portal phase (timer, 00:51) and mild washout in delayed phase.

As for 56 confirmed ICC by pathology, numbers of lesions classified as LR-3, LR-4, LR-5 and LR-M were 0, 0, 3 and 53, respectively on CEUS; while the corresponding numbers were 3, 0, 3 and 50 on CT/MRI. Thus, on CEUS, 94.6% of ICC could be correctly classified as LR-M, while 89.3% ICC could be classified as LR-M on CT/MRI. There was no significant difference in the sensitivity of LR-M category for ICC lesions between CEUS and CT/MRI LI-RADS (P = 0.375). Fifty ICC lesions (89.3%) were classified as the same LI-RADS categories by these two standardized algorithms (1 for LR-5 and 49 for LR-M, kappa value = 0.296, p = 0.002) (**Table 4**). A case of pathologically-confirmed ICC, which was correctly classified as LR-M both on CEUS and gadopentetate-enhanced MRI, was shown in **Figure 2**. Among 34 liver lesions confirmed as CHC by pathology, similar result was found between these two standardized algorithms (76.5% of CEUS and 70.6% of CT/MRI LIRADS, P = 0.754). Twenty-four lesions diagnosed as CHC by pathology (70.6%) were diagnosed as the same LI-RADS categories by two guidelines (4 for LR-5 and 20 for LR-M, kappa value = 0.264, p = 0.096) (**Table 4**). A case of CHC with different category on CEUS and gadopentetate-enhanced MRI was shown in **Figure 3**.

The inter-reader agreement was substantial for both CEUS and CT/MR (kappa value = 0.662, p <0.001 of CEUS; kappa value = 0.736, p <0.001 of CT/MRI), where no significant difference was found (p = 0.804).

## Discussion

High specificity of LR-5 for HCC and high sensitivity of LR-M for non-HCC malignancies are required to meet the purpose of LI-RADS on achieving accurate diagnosis, which helps to choose the proper treatments. Several previous studies have proved that there were no significant difference in the specificity of LR-5 for HCC between CEUS LI-RADS and CT/MR LI-RADS (18, 19, 21), however, studies directly focusing on the sensitivity of LR-M for non-HCC malignancies between these two algorithms remained limited. Thus, this retrospective study investigated the sensitivity of LR-M for non-HCC malignancies in a relatively large sample of non-HCC malignancies.

Our data implied that both CEUS LI-RADS v2017 and CT/MR LI-RADS v2018 showed substantial inter-reader agreement, which were consistent with that found by previous studies [0.76 of CEUS (22) vs 0.63 of CT/MRI (23)], suggesting that they are reliable algorithms to lead non-invasively distinguishing of non-HCC from HCC. The sensitivity of correctly classifying non-HCC primary hepatic malignancies as LR-M following the CEUS LI-RADS v2017 reached 88.3%, which was in accordance with the results of previous studies (sensitivity of LR-M, 81.1–97.3%) (15, 20, 24), validating the result of the prior studies that rim APHE, early-onset washout and marked washout were more frequently detected in ICCs than in HCCs no matter what the risk factor is (25–27). The sensitivity of LR-M category of CT/MRI according to the CT/MRI LI-RADS v2018 reached 81.9%, which was consistent with the previous studies (sensitivity of LR-M, 62–89%) (14, 28). The present study implied that the sensitivities of LR-M respectively following these two algorithms were comparable (p = 0.210). Thus, it is feasible to use the LR-M criteria of CEUS LI-RADS v2017 in distinguishing non-HCC from HCC in patients with risks factors for HCC. Although a recent study by Ding et al. (18) demonstrated that the sensitivity, specificity, and accuracy for diagnosing non-HCC malignancy were 90.9, 84.5, and 85.0% in CEUS LR-M and 63.6, 99.6, and 96.7% in CECT/MRI LR-M and drew the conclusion that CECT/MRI LR-M has better diagnostic performance for non-HCC malignancy than CEUS LR-M. We felt a little confused about the conclusion, not only because the sensitivity of LR-M contributes more larger than the specificity and accuracy in keeping non-HCC malignancy from LR-5 and avoiding improper treatments which threaten the overall survival (17), but also the small sample of non-HCC malignancy in that study made the conclusion less convincing.

The performances of these two algorithms for diagnosing ICC or CHC were also comparable in present study. The sensitivity of LR-M category of CT/MRI LI-RADS reached 89.3% for ICC and 70.6% for CHC, consisted with the previous studies [84% for ICC (29) and 61.4% for CHC (30)], while that of CEUS LI-RADS reached 94.6% for ICC and 76.5% for CHC, comparable to the prior studies [97.25% for ICC (20) and 83.3% for CHC (15)].

Our study also demonstrated that early-onset washout within 60 s (87.2%) was the most frequent feature found on CEUS for non-HCC malignancies, which was similar to the prior studies (91.9%) (31), while rim APHE was found in 28.7% of all 94 lesions that was relatively lower than that reported in most previous studies(38–69% of ICCs) (32, 33). This disaccord may have to do with differences between cohorts (non-HCC vs ICC) or the finding of the prior studies that most ICCs with cirrhosis or <30 mm with normal liver may display similar enhancement pattern to HCC (34, 35). As for CT/MR, rim APHE and delayed central enhancement were detected in 59.5% and 64.9% of all lesions, different from the prior studied (71% for rim APHE and 42% for delayed central enhancement) (14). The difference may be explained by different histological content within the cohorts that lesions with less peripheral vessel density and more fibrous stroma may prefer to present delayed central enhancement rather that rim APHE (36).

Interestingly, similar to the previous study (kappa value = 0.218 (37) for CEUS LI-RADS v2016 vs CT/MRI LI-RADS v2014; kappa value = 0.319 (18) for CEUS LI-RADS v2017 vs CT/MRI LI-RADS v2017; Spearman correlation coefficient scores = 0.546 (38) between the CT/MRI LI-RADS v2018 and CEUS LI-RADS v2017), inter-modality agreement was only fair for these two standardized algorithms (kappa value = 0.307), which could arise from the different working mechanism of contrasts used in the two methods. As is known, CT and MR contrast agents are small molecules that leak into the extracellular space of tumors resulting in prolonged enhancement of tumor while ultrasound contrast remains in the vessels during the vascular phases (39). Therefore, washout in CEUS, which reflects true contrast washout, cannot be equated with that in CT/MRI. Thus, different histological state of lesions, including arterial density, microvessel density, fibrous stroma and necrosis in the tumor, may make the lesion prefer to present a major feature of LR-M of only one algorithms but the other one (34, 35). Besides, we found that size measurement of nodules was of moderate agreement between these two algorithms. Difference in the nodule measured on its longest axis between CEUS and CT/MR in the present study may influent the consistence of category assigned by the two algorithms to some extent (38).

Several limitations of this study need to be declared. On the one hand, some features for categorization and influencing factors of the image quality were not evaluating in our study, such as threshold growth, depth of lesions and extent of cirrhosis or fatty liver, which may influent the final category. Besides, the current study lacked personal history and epidemiological data of patients, which should be acquired in the future study in order to recognize primary isolated hepatic tuberculosis, a mimic of hepatic malignancies in imaging diagnosis (40, 41). On the other hand, our findings may have limited generalizability due to small sample capacity enrolled from single center. However, our work was intended as a pilot study, which evaluates performance and inter-modality agreement of CEUS LI-RADS and CT/MRI LI-RADS in direct comparison on a relatively large sample of non-HCC malignancies.

In conclusion, our study demonstrates that CEUS LI-RADS v2017 and CT/MRI LI-RADS v2018 shows similar value for diagnosing non-HCC primary hepatic malignancies in patients with risks for HCC. However, further studies to enhance the inter-modality agreement between these two guidelines are needed.

## Data Availability Statement

The raw data supporting the conclusions of this article will be made available by the authors, without undue reservation.

## Ethics Statement

The studies involving human participants were reviewed and approved by Institutional review board of Sun Yat-sen University Cancer Center. Written informed consent for participation was not required for this study in accordance with the national legislation and the institutional requirements.

## Author Contributions

Study design: all authors. Collection data: R-SM, FL, S-YM, and Z-XG. Data analysis and interpretation: QL, JH, FL, S-YM, Y-XH, J-XS, and J-HZ. Manuscript writing: Y-XH. Final approval of manuscript: all authors. All authors contributed to the article and approved the submitted version.

## Conflict of Interest

The authors declare that the research was conducted in the absence of any commercial or financial relationships that could be construed as a potential conflict of interest.
